# Cotyledonary somatic embryo is one kind of intermediate material similar to callus in the process of in vitro tissue culture from *Rosa hybrida* ‘John F. Kennedy’

**DOI:** 10.1186/s12864-024-10256-8

**Published:** 2024-04-12

**Authors:** Li Du, Xiaoling Kang, Haoran Guo, Zhongfeng Zhu, Rui Wu, Meijing Yuan, Chuanyu Ding

**Affiliations:** 1 Analysis and Test Center, Nanyang Normal University, 473061 Nanyang, China; 2 Henan Province Engineering Research Center of Rose Germplasm Innovation and Cultivation Technique, Nanyang Normal University, 473061 Nanyang, China; 3 College of Life Science and Agricultural Engineering, Nanyang Normal University, 473061 Nanyang, China

**Keywords:** *Rosa hybrida* ‘John F. Kennedy’, Single-piece cotyledonary somatic embryo culture, Somatic embryogenesis and organogenesis, Seed propagation and vegetative propagation, Intermediate material in vitro tissue culture

## Abstract

**Background:**

Rose is recognized as an important ornamental plant worldwide, and it is also one of the most widely used flowers in gardens. At present, the improvement of rose traits is still difficult and uncertain, and molecular breeding can provide new ideas for the improvement of modern rose varieties. Somatic embryos are quite good receptors for genetic transformation. However, little is known about the molecular mechanisms underlying during the regeneration process of rose somatic embryos. To elucidate the molecular regulation mechanism of somatic embryo plantlet regeneration, the relationship between the differences in traits of the two different regenerated materials and the significantly differentially expressed genes (DEGs) related to phytohormone pathways in the process of regeneration were be investigated.

**Results:**

These representative two regenerated samples from single-piece cotyledonary somatic embryo (SPC) culture of *Rosa hybrida* ‘John F. Kennedy’, were harvested for transcriptome analysis, with the SPC explants at the initial culture (Day 0) as the control. The differentially expressed genes (DEGs) in the materials from two different types for regeneration approach (SBF type: the regeneration approach type of single bud formed from SPC explants; MBF type: the regeneration approach type of multiple buds formed from SPC explants) were be screened by means of the transcriptome sequencing technology. In this study, a total of about 396.24 million clean reads were obtained, of which 78.95–82.92% were localized to the reference genome, compared with the initial material (CK sample), there were 5594 specific genes in the material of SBF type and 6142 specific genes in the MBF type. The DEGs from the SBF type material were mainly concentrated in the biological processes of GO terms such as phytohormones, substance transport, cell differentiation, and redox reaction. The KEGG enrichment analysis revealed these DEGs were more active in ubiquinone and other terpenoid-quinone biosynthesis, fatty acid elongation, steroid biosynthesis, and glycosphingolipid biosynthesis-globo and isoglobo series. In contrast, the DEGs induced by the MBF type material were mainly associated with the biological processes such as phytohormones, phosphorylation, photosynthesis and signal transduction. According to KEGG analysis, these DEGs of MBF type were significantly enriched in the porphyrin and chlorophyll metabolism, brassinosteroid biosynthesis, carotenoid biosynthesis, and peroxisome. Furthermore, the results from the phytohormone pathways analysis showed that the auxin-responsive factor SAUR and the cell wall modifying enzyme gene *XTH* were upregulated for expression but the protein phosphatase gene *PP2C* was downregulated for expression in SBF type; the higher expression of the ethylene receptor ETR, the ethylene transduction genes *EBF1/2*, the transcription factor EIN3, and the ethylene-responsive transcription factor ERF1/2 were induced by MBF type.

**Conclusions:**

According to the GO and KEGG analysis, it indicated the DEGs between two different regenerated materials from somatic embryos were significantly different which might be causing morphological differences. That was somatic embryos from *Rosa hybrida* ‘John F. Kennedy’ could regenerate plantlet *via* both classic somatic embryogenesis (seed-like germination) and organogenesis, cotyledonary somatic embryos should be considered as one kind of intermediate materials similiar to callus, rather than the indicator materials for somatic embryogenesis.

## Background

Rose was the world’s most important ornamental plant [1]. Due to its characteristics of wide variety, long flowering period, extensive adaptability, and strong pruning resistance, rose was widely cultivated in China. There were so many varieties of modern rose that more than 25,000 new varieties had been cultivated in the past one or two hundred years, forming a huge group of hybrid varieties of modern rose. However, it was still difficult and uncertain to develop the flower color and stress resistance for modern rose by means of traditional hybrid breeding method [2]. Molecular breeding has opened new ways to improvement of modern rose. Establishment of an efficient plant regeneration system and genetic transformation system was a necessary condition for molecular breeding.

Under in vitro conditions, plant could regenerate through two pathways, somatic embryogenesis and organogenesis [3]. Somatic embryogenesis is the process that plant somatic cells or tissues as explants can develop complete plants through the developmental program of zygotic embryos, under suitable culture conditions. For example, while the leaves of *Rosa hybrida* ‘Samantha’ [4] and cotyledons in the tea oil plant (*Camellia oleifera* Abel.) were selected as the explants for somatic embryos induction [5], it could obtain the regenerated plants of the two species through root and bud development synchronization from somatic embryos (seed-like germination). It is usually considered that somatic embryos have bipolarity, and they are the indicative materials for somatic embryogenesis, namely, once the observation of somatic embryos formation rather than somatic embryos plantlet regeneration like seeds, the culture results can be already seen as somatic embryogenesis. Additionally, previous studies had shown that organogenesis was the process that plant explants could differentiate adventitious buds or roots along only one direction in vitro culture, and then these two structures were connected through vascular bundles to form a complete plant. As cotyledonary node of Peanut (*Arachis hypogaea* L.) and tuber discs from yams (*Dioscorea tokoro* Makino) being explants [6–7], all could be induced callus, then the adventitious buds could be derived from the callus after the root formation from the rootless seedlings, these species could obtain the complete regenerated plants. Plantlet regeneration approaches (somatic embryogenesis or organogenesis) could be divided into direct and indirect approach based on whether or not the formation of callus, the callus was regarded as the intermediate material in vitro culture [8–11]. The regeneration pathway of somatic embryogenesis was considered to be of single-cell origin, with the advantages of fewer chimeras and consistent genetic background [12–13]. Somatic embryos were often used as excellent receptors in plant molecular breeding, and the molecular mechanism of somatic embryogenesis had also received significant attention from researchers [14–15].

Transcriptome sequencing was a technique that used high-throughput sequencing approaches to determine the sequence of all RNA transcripts in a given sample, which could obtain a large amount of genetic information from different species [16]. Many meaningful research results had been obtained in the exploration of potential functional genes [17], analysis of traits [18, 20] and physiological regulation mechanisms in plants [19] by high-throughput transcriptome sequencing technology, which was also often used to analyze the relevant mechanisms of plant development processes [15, 21]. Tang et al. [17] used transcriptome sequencing data to investigate the reason of character differences of Shatang tangerine (*Citrus reticulata*) and Lantern Shatang tangerine, and screened out key DEGs related to wax biosynthesis (*CER1*, *CER3*, *HHT*, cytochrome *P45086A8*, and cytochrome *P45086A22*) that might cause wax loss on the surface of Lantern Shatang tangerine. Transcriptome sequencing was performed on the branches of the golden type and common type from *Salix caprea* var. *aurea*, and it was showed that the expression levels of the genes related to chlorophyll synthesis was substantially changed, leading to affected the metabolic pathways, resulting in significant differences in the color of branches [18]. Recent studies had found that most DEGs involved in photosynthesis were upregulated in black point-slightly susceptible lines, indicating that photosynthesis played a crucial role in regulating wheat resistance to black point [19]. Transcriptome analysis of Neptune King rose and its color mutant, Queen rose at the blooming stage revealed that, fifteen genes participating in the flavonoid pathway and thirty transcription factors, were differentially expressed between the two cultivars. It is worth noting that thirteen anthocyanin biosynthetic related genes showed an upregulation trend in the mutant flower, which may favor the higher levels of anthocyanins in the mutant [20].

Significantly, in cotton, purine metabolism played an important role in embryogenic callus proliferation, and the flavonoids biosynthesis might be closely related to somatic embryo development [15]. A large number of genes related to plant hormones, transcription factors, and stress response were differentially expressed during the induction of somatic embryos from the *Rosa hybrida* ‘Baisemeian’ [21]. Moreover, the DEGs of the high-regeneration lines were mainly related to taurine and hypotaurine metabolism, nitrogen metabolism, and fatty acid elongation, while those of the low regeneration lines were involved in photosynthesis, porphyrin and chlorophyll metabolism, and plant hormone signal transduction. This study could provide new idea for elucidating the molecular regulatory mechanisms of early maize redifferentiation [22]. It could be seen that transcriptome sequencing technology had important application value in exploring the process of somatic embryogenesis and analyzing the regulatory mechanism of somatic embryo regeneration.

In order to explore the process of plant regeneration from somatic embryos, the different types of regenerated materials from single-piece cotyledonary somatic embryo (SPC explants) culture of *Rosa hybrida* ‘John F. Kennedy’, with the initial somatic embryos as the control, were used for sequencing. By using high-throughput sequencing technology for transcriptome sequencing, the relationship between the differences in traits of the two types of regenerated materials and the significantly differentially expressed genes (DEGs) related to phytohormone pathways during the process of regeneration would be surveyed. Finally, by identifying the gene expression characteristics of two regenerated materials with different traits and analyzing the molecular regulation mechanism of somatic embryo plantlet regeneration, the function and status of somatic embryo in the process of plantlet regeneration in vitro culture were clarified.

## Results

### Single-piece cotyledonary somatic embryo culture of *Rosa hybrida* ‘John F. Kennedy’

An efficient *Rosa hybrida* ‘John F. Kennedy’ somatic embryo (SE) system was established based on our previous approach as published recently, and different embryogenic cultures could be obtained by same one tissue culture experiment under same initial material and culture conditions [23].

**Fig. 1:**
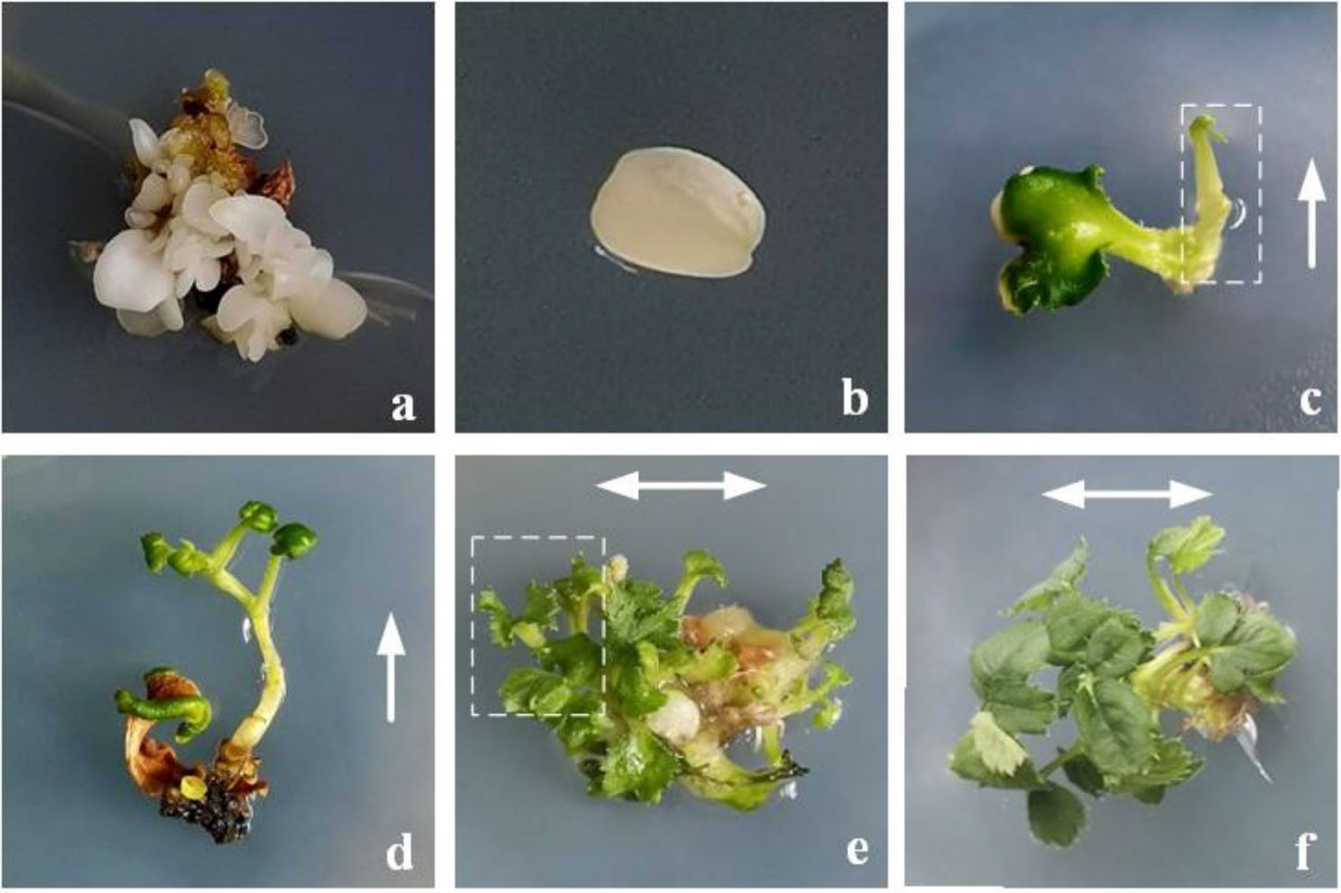
Different regeneration results during the SPC culture of *Rose hybrida* ‘J. F. Kennedy’. (**a**) Cotyledonary somatic embryos (CSE) propagation; (**b**) Single-piece cotyledonary somatic embryos explants (SPC initial material); (**c**) Single bud formation from SPC explants (SBF type material); (**d**) The regenerated seedling *via* SBF approach; (**e**) Multiple buds formation from SPC explants (MBF type material); (**f**) Multiple buds proliferation *via* MBF approach

After cotyledonary somatic embryos (CSE) were subcultured on P medium for 30 days, clusters of somatic embryos propagation could be observed (Fig. 1a). Complete single-piece cotyledonary somatic embryos explants (SPC explants, Fig. 1b) were selected from proliferated CSE and were incubated to P medium, after 35 days of culture, two different culture results could be observed (Fig. 1).

Single bud formed at the base of SPC explants (SBF type, Fig. 1c), and continued to culture, the single bud grew along the longitudinal axis and became strong (Fig. 1d). The way to regenerate a single bud from SPC explants could be considered as SBF regeneration approach. In addition, it could also be observed that buds could be formed at any position from the explants (MBF type, Fig. 1e). Generally, these regenerating buds appeared as multiple buds and proliferated along the horizontal axis (Fig. 1f), and the way to regenerate multiple buds from SPC explants could be considered as MBF regeneration approach.

When SBF buds or MBF buds were cut and transferred to P medium, all the buds could propagate and develop, and the young shoots could produce complete plantlets on MS medium. Furthermore, there was no significant difference in morphological characteristics of the regenerated plantlets whether *via* SBF approach or *via* MBF approach.

During the process of tissue culture for SPC explants of *Rosa hybrida* ‘John F. Kennedy’, the cultured materials could be classified into two types according to their external morphological characteristics and endogenous hormone content [24], in which the materials from two different regeneration types were single bud formed from SPC explants (SBF type, Fig. 1c) and multiple buds formed from SPC explants (MBF type, Fig. 1e) after 35 days of SPC explants culture. These representative samples from different SPC culture were harvested and pooled together or maintained separately for transcriptome analysis, with the SPC explants (CK, Fig. 1b) at the initial culture (Day 0) as the control.

### Transcriptome profiling of the two regenerated materials

After filtering low-quality reads and aptamer-contaminated reads, transcriptome sequencing of the nine libraries produced a total of 59.45 Gb clean reads, with more than 94% of the bases scoring Q30 (Table 1). The matching rate between clean reads and reference genome (Rosa chinensis RchiOBHm-V2) ranged from 78.95 to 82.92% (Table 1). 78.11-80.54% of the reads could be uniquely mapped to the reference genome sequence, indicating that the sequencing results had a high comparison rate and relatively perfect assembly, which can ensure the accuracy of the data.

**Table 1:** Data quality control and assembly quality data statistics

Sample	Raw Date Base	Valid Date Base	Valid Ratio Reads	Q20%	Q30%		Mapped Reads	Unique Mapped reads	
CK_1	5.89G	5.75G	97.67	99.95	95.56	81.27%			79.04%
CK _2	7.74G	7.56G	97.69	99.95	95.55	82.49%			80.23%
CK _3	6.80G	6.65G	97.71	99.93	94.64	80.53%			78.11%
SBF_1	7.51G	7.33G	97.56	99.96	95.82	82.92%			80.54%
SBF _2	5.85G	5.70G	97.41	99.94	94.70	79.40%			79.40%
SBF _3	5.06G	4.94G	97.52	99.93	94.65	78.95%			78.95%
MBF_1	7.88G	7.70G	97.66	99.95	95.54	82.03%			79.54%
MBF _2	7.07G	6.90G	97.65	99.93	94.62	81.03%			78.65%
MBF _3	7.09G	6.92G	97.68	99.95	95.02	81.57%			79.16%

**Fig. 2:**
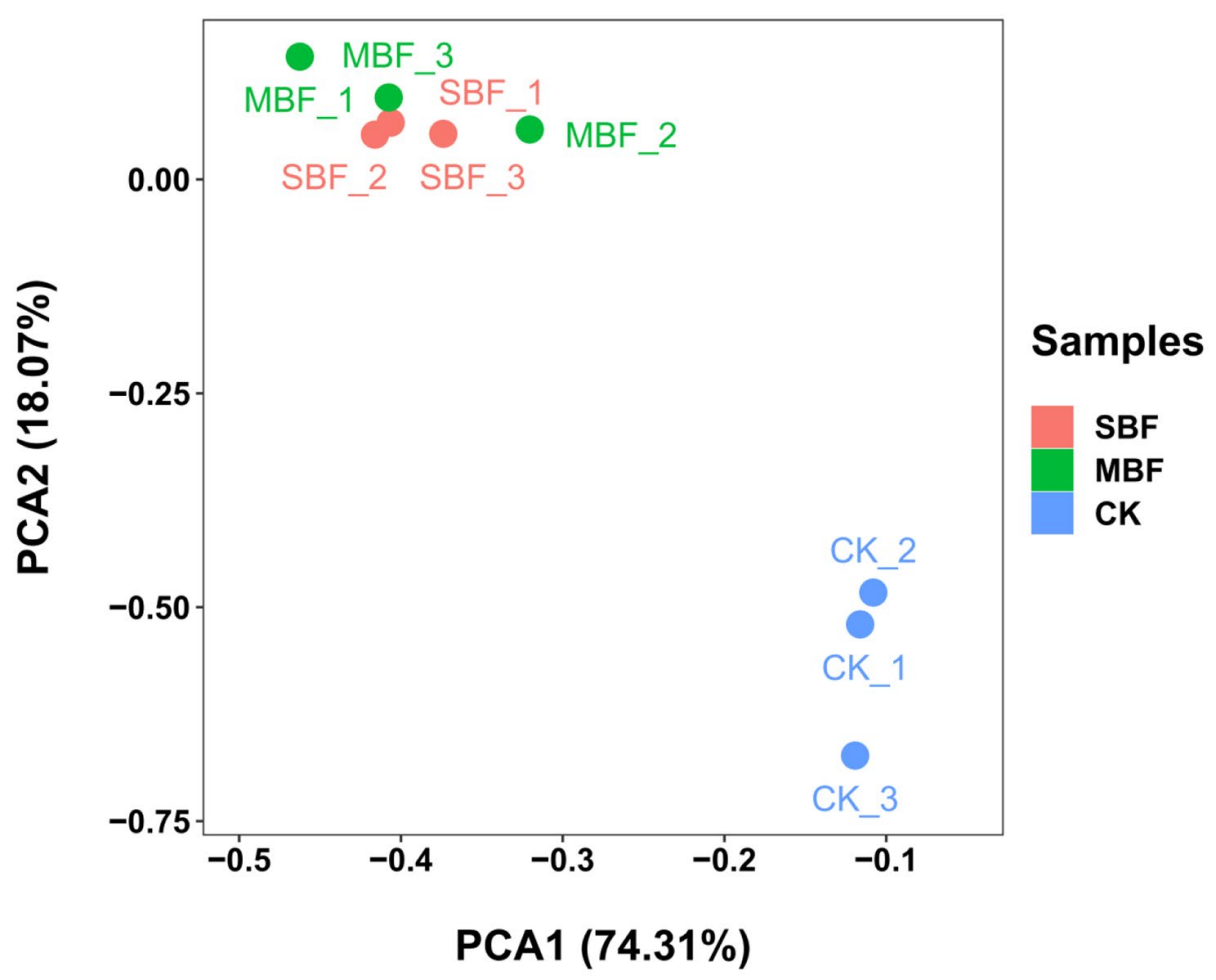
Principal components analysis of the three samples

In order to obtain the differentially expressed genes (DEGs) accurately, this study was performed by principal component analysis (PCA) analysis and Pearson correlation coefficient determination to understand the duplication between samples. PCA determined that the contribution rates of PCA1 and PCA2 were 74.31% and 18.07%, respectively. The three samples were obviously separated, and each formed a cluster (Fig. 2). Transcriptome based PCA of the date revealed significant differences between the three samples. Pearson correlations between biological replicates exceeded 0.90 in all pairwise comparisons, demonstrating that the results were highly reproducible (Table 2).

**Table 2:** Pearson correlation statistics between biological replicates

Sample 1	Sample 2	Pearson Correlation Coefficient
CK_1	CK _2	0.978
CK _2	CK _3	0.914
CK _3	CK _1	0.922
SBF_1	SBF _2	0.955
SBF _2	SBF _3	0.937
SBF _3	SBF _1	0.951
MBF_1	MBF _2	0.937
MBF _2	MBF _3	0.938
MBF _3	MBF _1	0.957

**Fig. 3:**
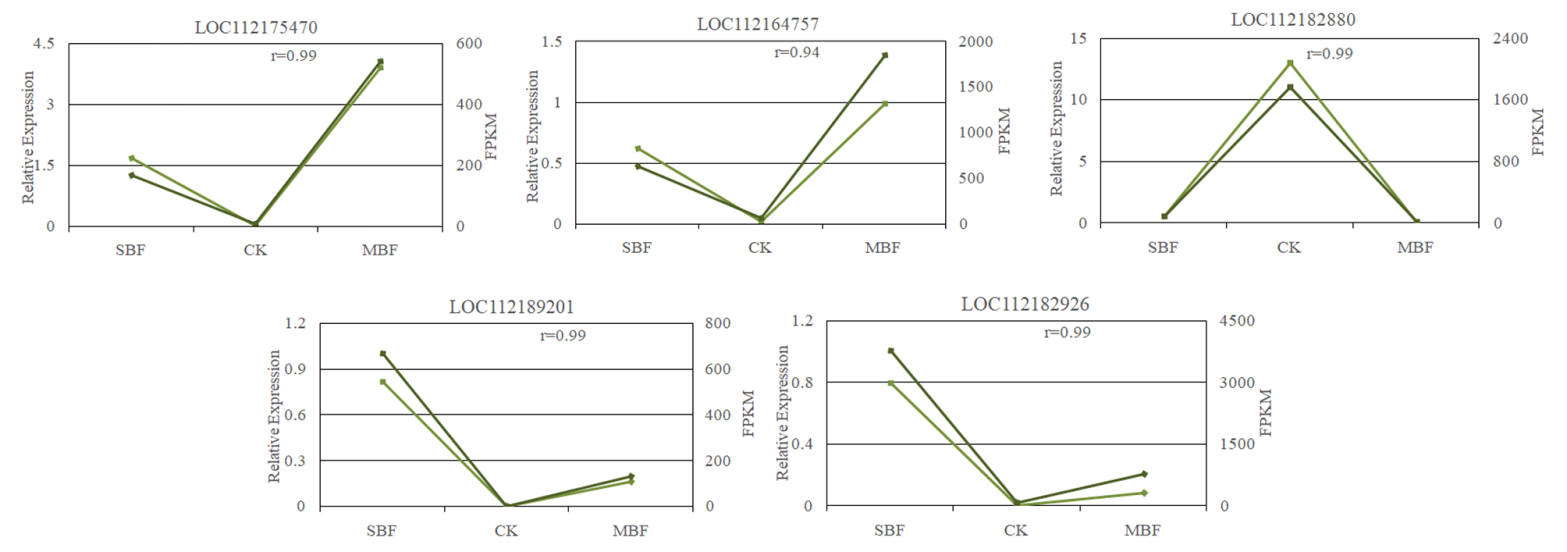
Correlation of differential expression rates between RNA-Seq and qRT-PCR for five DEGs. The comparison of RNA-Seq and qRT-PCR data assays was calculated with the pearson correlation and expressed by the r value. The left y-axis scale represented log2 of the relative expression level value from RT-qPCR. The righty-axis scale represented expression level from PCR-seq data

The expression levels of five randomly selected genes were confirmed by quantitative real-time PCR analysis and with characterized differently in different samples. The analysis results showed that the relative expressions of the five genes in three samples were consistent with the trend of transcriptome sequencing results, and the Pearson’s correlation coefficients between the relative expressions and the transcriptome sequencing data were all greater than 0.9, indicating that the RNA-Seq data were reliable (Fig. 3).

### DEGs identification across two regenerated materials

**Fig. 4:**
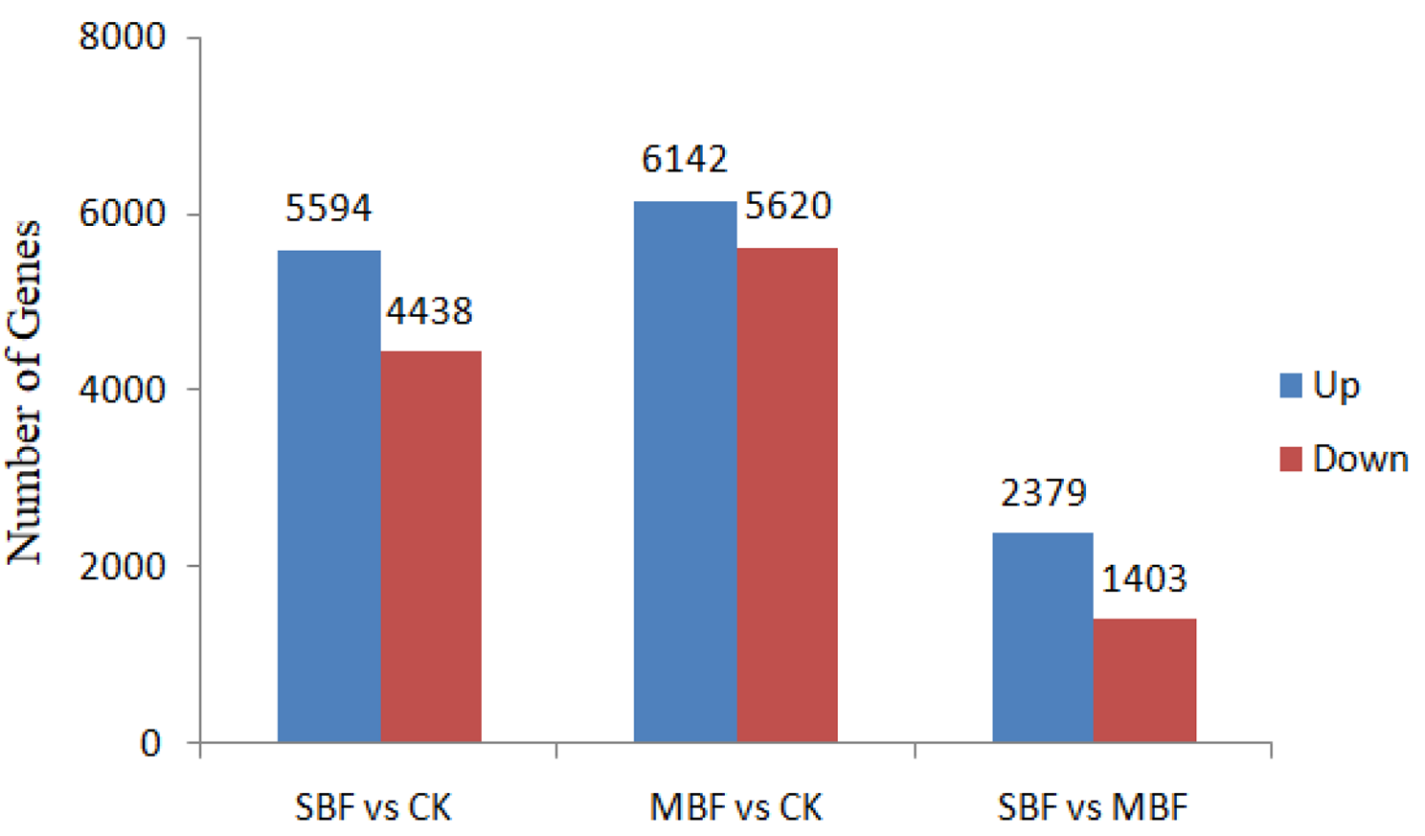
Histogram of the number of differential genes in different comparison groups

The results of the analysis based on principal component analysis and Pearson’s correlation coefficient showed that the sequencing data of the three samples had good biological reproducibility. The DEGs with upregulated and downregulated expression in the three comparison groups (SBF vs. CK, MBF vs. CK and SBF vs. MBF) were screened, utilizing the evaluation criteria of log_2_FC ≥ 1 and q < 0.05, log_2_FC ≤ 1 and q < 0.05, respectively, and the histogram of the number of DEGs was generated to compare the number of genes in the three groups (Fig. 4). A total of 10,032 DEGs, including 5,594 upregulated and 4,438 downregulated DEGs were identified between SBF material and CK sample (Fig. 4). The comparison between the MBF material and CK sample had the largest number of DEGs (11,762), which reflected the largest phenotypic variation between materials. Among these DEGs, 6,142 were upregulated with higher expression and 5,620 were downregulated in MBF material relative to CK sample (Fig. 4). Also, there were 3,782 genes uniquely present in SBF material and MBF material, of which 2,379 were upregulated and 1,403 were downregulated (Fig. 4).

### GO functional annotation and KEGG enrichment analysis of DEGs

To further understand the properties and biological functions of the DEGs in the three samples, the upregulated and downregulated DEGs in SBF vs. CK and MBF vs. CK were selected for GO functional annotation and KEGG enrichment analysis, respectively.

### GO functional annotation of DEGs

**Fig. 5:**
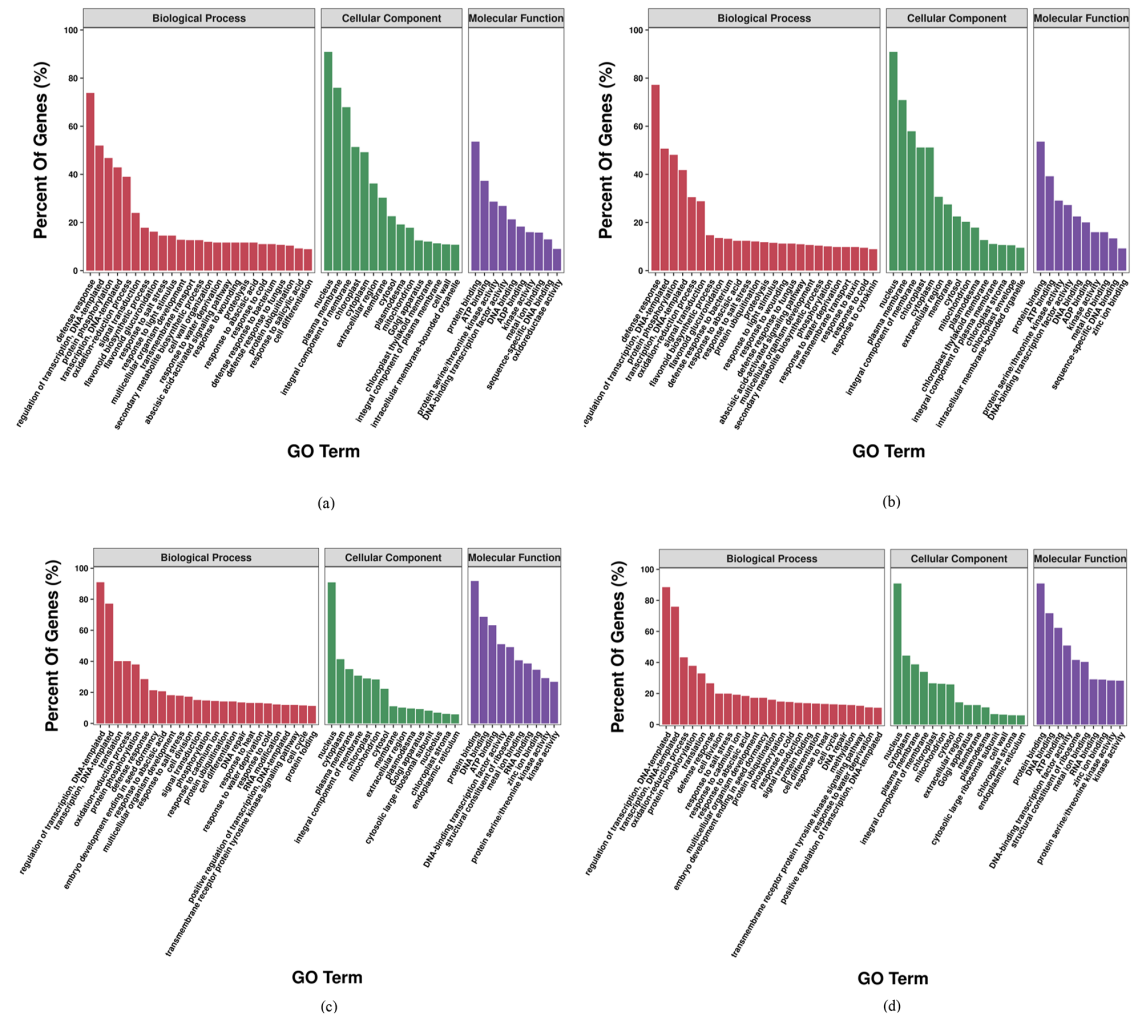
GO functional annotation of DEGs in two comparison groups. (**a**) GO function enrichment of upregulated genes in SBF and CK; (**b**) GO function enrichment of upregulated genes in MBF and CK; (**c**) GO function enrichment of downregulated genes in SBF and CK; (**d**) GO function enrichment of downregulated genes in MBF and CK

GO had three gene ontologies, including biological process (BP), cellular component (CC) and molecular function (MF). Among the upregulated DEGs in SBF vs. CK, 5,058 were assigned with functional annotations and were efficiently annotated to 2644 GO terms, involving 1,473 terms for BP, 249 terms for CC, and 922 terms for MF (Fig. 5a), and 3,665 downregulated DEGs were annotated with functions, including 1,552 terms in BP, 393 terms in BP and 926 terms in BP (Fig. 5c). There were 5,384 upregulated DEGs in MBF vs. CK that were effectively annotated to 2,850 GO terms, including 1,577 terms for MF, 282 terms for CC, and 991 terms for BP (Fig. 5b). As for the downregulated DEGs, 4768 DEGs had functional annotations with 1,722 GO terms in BP, 425 GO terms in BP and 1,048 GO terms in BP (Fig. 5d). The terms of BP, CC and MF were sorted from largest to smallest according to the percent of genes, and the terms of Top25, Top15 and Top10 were selected to draw the histogram of GO enrichment classification (Fig. 5).

The upregulated DEGs in the two comparison groups were collectively involved in biological processes such as defense response, regulation of transcription, DNA-templated, protein phosphorylation, oxidation-reduction process, signal transduction and flavonoid biosynthetic. In terms of CC, the identified genes were mainly associated with the nucleus, plasma membrane, chloroplast, cytoplasm, mitochondrion and chloroplast thylakoid membrane. In MF, some GO terms had higher enrichment frequencies, including protein binding, ATP binding, protein serine/threonine kinase activity, sequence-specific DNA binding and DNA-binding transcription factor activity. It was worth noting that the specific genes of the SBF material were individually included in BP for cell wall organization, response to salicylic acid, and cell differentiation, which were located in the cellular components of the Golgi apparatus and the cell wall, and were involved in the molecular function of oxidoreductase activity (Fig. 5a). The specific genes of the MBF material were mainly involved in biological processes such as phosphorylation, response to auxin, and response to cytokinin, followed by cellular components, the DEGs were mainly located in the chloroplast envelope and the chloroplast stroma, and their molecular functions were related to zinc ion binding (Fig. 5b).

The downregulated DEGs in the two comparison groups showed higher expression levels in the cotyledonary somatic embryo (initial material). By analyzing and comparing the 100 GO terms annotated by these downregulated genes, the similarity of GO terms reached 96.00% (Fig. 5c and d). Importantly, the DEGs of the initial material and the two regenerated materials (materials for SBF and MBF) were involved in three gene ontologies of BP, CC, and MF, with the greatest differences in BP, where 72% of the GO terms were different. However, in CC and MF, 26.67% and 30% of GO terms categories were different, respectively (Fig. 5). The DEGs regulating the two regenerated materials were commonly involved in 8 BP (flavonoid biosynthetic process, flavonoid glucuronidation, secondary metabolite biosynthetic process, abscisic acid-activated signaling pathway, response to wounding, proteolysis, defense response to fungus, and defense response to bacterium), 3CC (chloroplast thylakoid membrane, intracellular membrane-bounded organelle, and chloroplast stroma) and 4 MF (ADP binding, sequence specific DNA binding, and oxidoreductase activity, and zinc ion binding) (Fig. 5a and b). However, these GO terms were not found in the initial material. The genes regulating the initial material were involved in 8 terms for BP, 3 terms for CC, and 2 terms for MF. BP included embryo development ending in seed dormancy, response to cadmium ion, DNA repair, response to heat, RNA modification, positive regulation of transcription, DNA-templated, transmembrane receptor protein tyrosine kinase signaling pathway, and protein folding; CC involved cytosolic large ribosomal subunit, nucleolus, and endoplasmic reticulum; Meanwhile, MF included the structural constituent of ribosome and RNA binding (Fig. 5c and d). But these GO terms were not annotated in the two regenerated materials.

According to the analysis of GO, compared with the initial material, the specific genes of the two regenerated materials were categorized into three functions, showing significant differences, indicating that the genes regulating the two kinds of culture results (materials for SBF and MBF) had different attributes. Therefore, it is speculated that these two regenerated materials might be obtained by the different plant regeneration approaches.

### KEGG enrichment analysis of DEGs

**Fig. 6:**
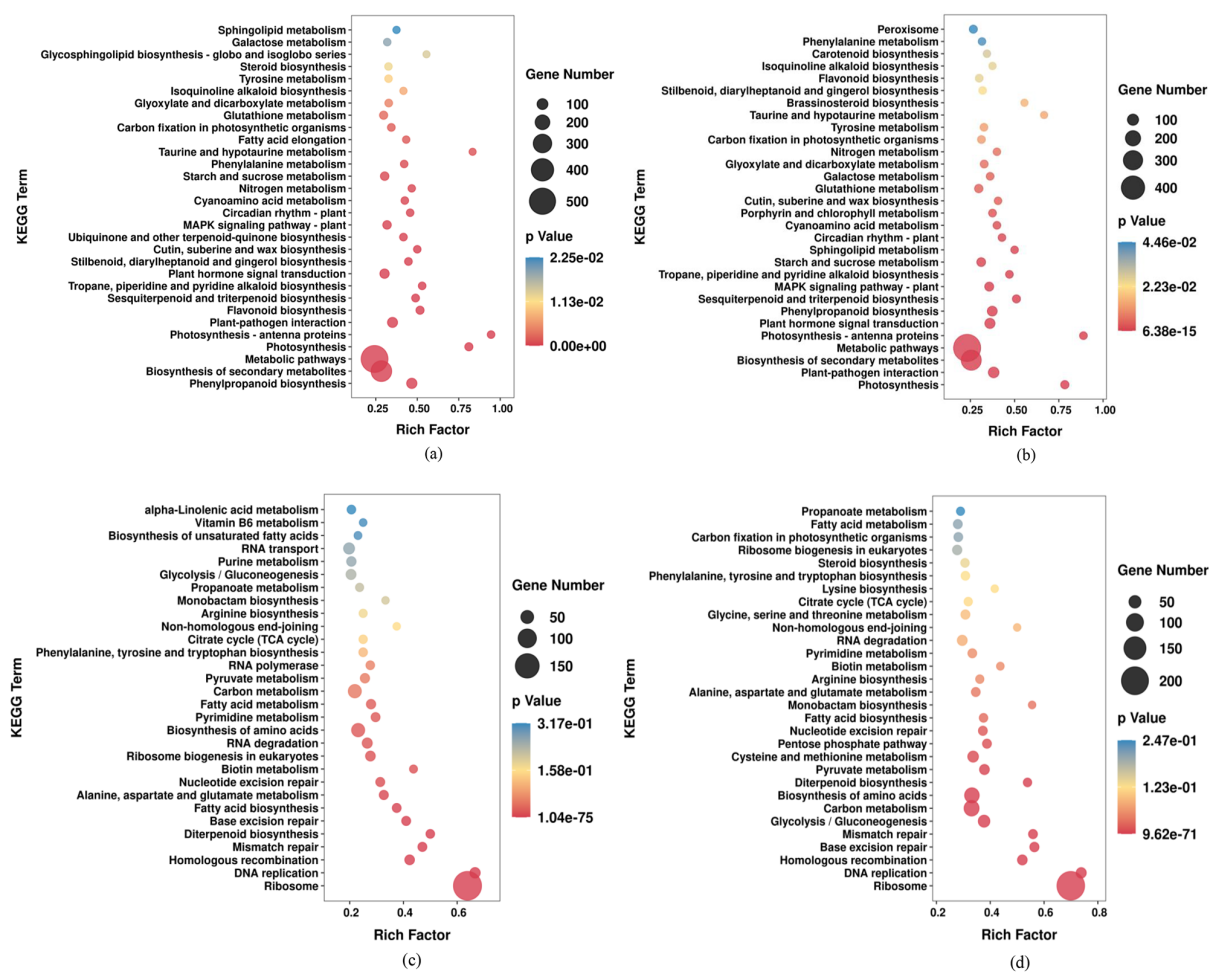
KEGG enrichment analysis of DEGs in two comparison groups. (**a**) Pathway enrichment of upregulated genes in SBF and CK; (**b**) Pathway enrichment of upregulated genes in MBF and CK; (**c**) Pathway enrichment of downregulated genes in SBF and CK; (**d**) Pathway enrichment of downregulated genes in MBF and CK

In organisms, different genes coordinate with each other to perform their biological functions. For further understanding the biological functions of DEGs, the pathways that were significantly enriched compared with the whole genomic background were screened and analyzed. In SBF vs. CK, it was found that 5060 upregulated DEGs and 3665 downregulated DEGs were involved in 188 metabolic pathways, and the significantly enriched metabolic pathways (*P*-value ≤ 0.05) had 36 and 17, respectively. In MBF vs. CK, 5388 upregulated DEGs and 4768 downregulated DEGs with functional annotations affected 125 and 122 metabolic pathways, and the significantly enriched metabolic pathways had 31 and 18, respectively. Based on the filtering results, KEGG pathway enrichment bubble plots were drawn using ggplot2, and the Top30 pathways with the minimum *P*-value were selected for mapping and display (Fig. 6).

Among the upregulated genes in the two groups, numerous DEGs were involved in the pathway of metabolic pathways and biosynthesis of secondary metabolites, the photosynthesis-antenna proteins and photosynthesis were the most enriched metabolic pathways. In addition, 22 common enrichment pathways (plant-pathogen interaction, flavonoid biosynthesis, plant hormone signal transduction, circadian rhythm-plant, starch and sucrose metabolism, taurine and hypotaurine metabolism, carbon fixation in photosynthetic organisms, tyrosine metabolism and galactose metabolism) were shared by the two regenerated materials (materials for SBF and MBF). However, these metabolic pathways were different in the enrichment significance, degree of enrichment and number of enrichment genes between the two regenerated materials (Fig. 6a and b). These metabolic pathways played a conserved and important role in the regeneration process of *Rosa hybrida* ‘John F. Kennedy’.

In SBF vs. CK, 85 DEGs were involved in the pathway of phenylpropanoid biosynthesis, which was the most significant upregulated metabolic pathway enriched in the SBF type. These genes regulating the growth and development of the SBF material were individually assigned to four metabolic pathways, including ubiquinone and other terpenoid-quinone biosynthesis, fatty acid elongation, steroid biosynthesis, and glycosphingolipid biosynthesis-globo and isogloboseries (Fig. 6a). Photosynthesis was the most significantly enriched metabolic pathway for the 29 upregulated DEGs by the MBF type. For the genes regulating the MBF type, they were separately involved in the metabolic pathways of porphyrin and chlorophyll metabolism, brassinosteroid biosynthesis, carotenoid biosynthesis, and peroxisome (Fig. 6b).

The downregulated DEGs in the two comparison groups were specific genes in the initial material, and the similarity of enrichment pathways accounted for 80.00%, including alanine, aspartate and glutamate metabolism, biotin metabolism, biosynthesis of amino acids, pyrimidine metabolism, pyruvate metabolism, the TCA cycle, and glycolysis/gluconeogenesis (Fig. 6c and d). In SBF vs. CK, the specific DEGs of the initial material (Fig. 6c) and the SBF material (Fig. 6a) were involved in 30 metabolic pathways, respectively, of which 28 were different; however, 29 metabolic pathways were different between the two materials in MBF vs. CK (Fig. 6b and d).

The results of KEGG analysis detected that the specific genes of the two regenerated materials were significantly included in different metabolic pathways categories, compared with the initial material, implying that in this study, the two culture results obtained from the culture of rose single-piece cotyledonary somatic embryo were regulated by different metabolic pathways. In addition, there were eight different metabolic pathways between two regenerated materials; it was evident that different metabolic pathways might have determined the SBF regeneration approach different from MBF regeneration approach of the cotyledonary somatic embryo culture of *Rosa hybrida* ‘John F. Kennedy’.

### Mechanisms of plant hormone signaling

**Fig. 7:**
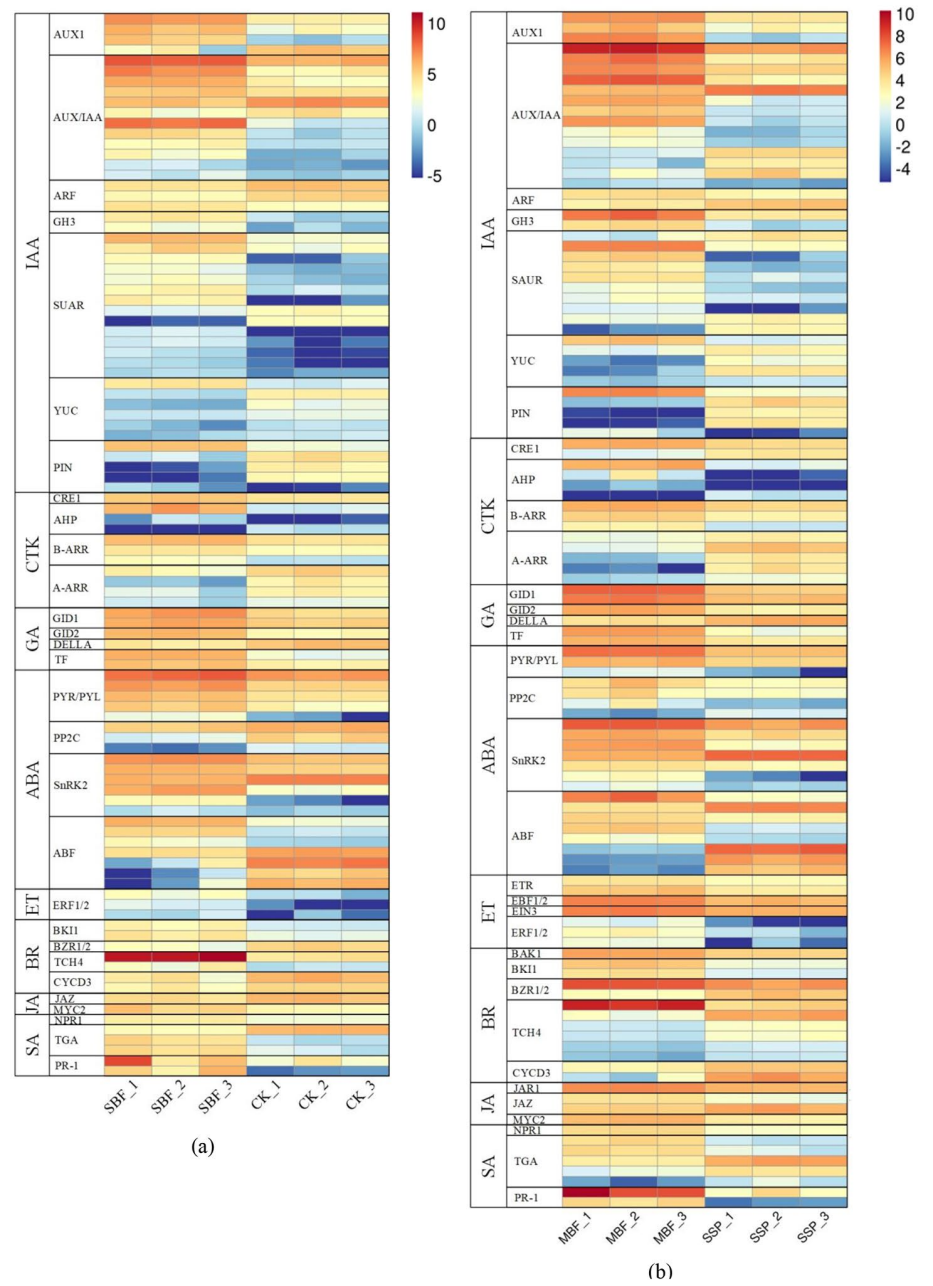
The relative expression pattern of DEGs involved in plant hormone signal transduction mechanisms. (**a**) Relative expression patterns of deg involved in phytohormone signaling mechanisms in SBF vs. CK; (**b**) Relative expression patterns of deg involved in phytohormone signaling mechanisms in MBF vs. CK

Phytohormones played crucial roles in regulating plant growth and development. In this study, we observed that the DEGs of the two regenerated materials were included in the plant hormone pathway. In order to explore hormone-mediated transcriptional regulation in gene expression during the process of tissue culture for SPC explants of *Rosa hybrida* ‘John F. Kennedy’, the DEGs were mapped to eight hormone-related pathways (auxin, cytokinin, abscisic acid, brassinosteroid, ethylene, gibberellin, jasmonic acid, and salicylic acid) and these genes were related to hormone biosynthesis, response, signaling, receptors, and metabolism. The expression levels of the above genes had been significantly different in these two regenerated materials (Fig. 7).

In this research, three samples of *Rosa hybrida* ‘John F. Kennedy’ collected the most number of significantly DEGs in the auxin-related pathway, including *YUCCA* (YUC) genes involved in auxin synthesis, *PIN-FORMED (PIN)* and *AUXIN1 (AUX1)* members included in auxin transport, *auxin/indole-3-acetic acid (AUX/IAA)*, *Gretchen Hagen3 (GH3)*, and *Small auxin-up RNA (SAUR)* involved in auxin response. Within the auxin pathway, we found the *YUC* and *PIN* genes, which were highly upregulated in the cotyledonary somatic embryo (initial material) of *Rosa hybrida* ‘John F. Kennedy’. Compared with the initial material, *AUX1*, *GH3*, and *SAUR* genes were significantly upregulated in the two regenerated materials (materials for SBF and MBF), all of which were downregulated in the initial material (Fig. 7a and b). Furthermore, our results show that the majority of the *SAUR* genes were dramatically upregulated in the SBF material to regulate its growth and development (Fig. 7a). Moreover, the expression of the cytokinin pathway genes was altered in three sample materials. For example, 2 histidine kinase (CRE1) genes, including *HK3*, and *HK4*, were upregulated in the two regenerated materials. All two-component response regulators (type-A ARRs) of the cytokinin signaling pathway were substantially upregulated in the initial material, but downregulated expression in the two regenerated materials, in contrast to type-B response regulators (type-B ARRs) (Fig. 7a and b).

Abscisic acid (ABA) is a key hormone for plant growth, development, and stress acclimation, and can affect plant stomatal closure and seed dormancy. In this study, ABA signal transduction pathway included four core components namely PYR/PYL receptors, type 2 C protein phosphatase (PP2Cs), serine/threonine-protein kinase (SnRK2s), and transcription factor (ARF). Compared to the initial material, all 3 *PP2C* genes identified in the rose transcriptome were significantly downregulated in the SBF material and 75% of the *PP2C* genes were upregulated in the MBF material (Fig. 7a and b). Related with ethylene-related pathway, we identified four components, including ethylene receptor protein ETR, ethylene signaling genes *EBF1/2*, transcription factor EIN3, and ethylene-responsive factors ERF1/2, which were highly expressed in the MBF material (Fig. 7b). However, only the expression of ethylene response factor (ERF1/2) was slightly upregulated in the SBF material (Fig. 7a).

Additionally, the expression levels of the genes related with the BR signaling pathway was substantially altered between the initial material and two regenerated materials. Compared with the initial material, most of the genes *(BKI1, BZR1/2, TCH4 and CYCD3)* were upregulated in the SBF material (Fig. 7a and b). Among them, the cell wall modifying enzyme genes *(XTHs)* involved in ‘cell wall’ were dramatically enriched in the SBF material (Fig. 7a). In SBF vs. MBF, all 13 *XTH* genes identified in the rose transcriptome were markedly upregulated, and it will be entertaining to research their roles in the SBF regeneration approach (seed-like germination) of *Rosa hybrida* ‘John F. Kennedy’ in the future. The above results indicate that, the *XTH* genes of the BR signaling pathway might be the key regulating the growth and development of the SBF material of *Rosa hybrida* ‘John F. Kennedy’. This class of genes could alter the extensibility of the cell wall, causing stem cells of the SBF material to elongate and show a longitudinal growth trend, eventually regenerating through seed-like germination.

## Discussion

### Two different plantlet regeneration approaches of somatic embryo from *Rosa hybrida* ‘John F. Kennedy’

In our previous study and this research, it observed that the cotyledonary somatic embryos of *Rosa hybrida* ‘John F. Kennedy’ could regenerate plantlets by means of two different approaches [23]; there were single-bud-formation regeneration (SBF) and multiple-buds-formation (MBF) regeneration, respectively. SBF regeneration could be observed that a single bud induced from SPC explant usually, and the seedling from single bud grew and became strong along the longitudinal axis (Fig. 1c and d). Another approach was MBF regeneration, which could be observed adventitious buds derived from SPC explant, and the multiple buds proliferated along the horizontal axis (Fig. 1e and f).

In this study, transcriptome sequencing was utilized to screen for significantly differentially expressed genes in two different regenerated materials (SBF type and MBF type) that obtained from SPC culture of *Rosa hybrida* ‘John F. Kennedy’. Compared to the initial material (SPC explant), DEGs induced by SBF material were mainly concentrated in GO terms of phytohormones, substance transport, cell differentiation and redox reaction, and KEGG enrichment analysis revealed that they were mainly associated with ubiquinone and other terpenoid-quinone biosynthesis, fatty acid elongation, steroid biosynthesis, and glycosphingolipid biosynthesis-globo and isoglobo series. In contrast, the DEGs from MBF material were mainly associated with the biological processes such as phytohormones, phosphorylation, photosynthesis and signal transduction. According to KEGG analysis these DEGs were significantly included in the porphyrin and chlorophyll metabolism, brassinosteroid biosynthesis, carotenoid biosynthesis, and peroxisome.

According to the GO and KEGG pathway enrichment analysis, it indicated that the DEGs of these genes in biological processes and in metabolic pathways might be causing morphological differences between the two regenerated materials, and then promoting somatic embryo explant to regenerate plantlet under different approaches. In this study, SBF approach and MBF approach of *Rosa hybrida* ‘John F. Kennedy’ would be considered as seed-like germination (classic somatic embryogenesis), and organogenesis from somatic embryo explants, respectively.

### Plantlet regeneration approaches and phytohormone pathways

In this study, the specific common DEGs from the initial material of somatic embryo explant and the two regenerated materials (materials for SBF and MBF) were mainly associated with the phytohormone metabolic pathways of auxin, cytokinin, brassinosteroid and ethylene.

In the previous studies about auxin metabolic pathways, the auxin polarity transport factor *PIN1* was an essential gene for somatic embryo formation [25], and the *YUCCA* family genes related with auxin biosynthesis seemed as key enzymesin the regulation of late stage of somatic embryo [26–27]. Most *PIN* and *YUC* genes were significantly upregulated in the initial material of *Rosa hybrida* ‘John F. Kennedy’, it suggested that *YUC* and *PIN* also might be key genes affecting somatic embryos induction and propagation in this study. Among genes of auxin transactivation, it had been reported that the role of *SAUR* genes in plant growth is mainly characterized by the promotion of cell elongation, and SAUR proteins could also induce cell elongation on a plant-wide scale by inhibiting the activity of *PP2C* [28]. Notably, most of the *SAUR* genes were upregulated and all the protein phosphatase *PP2C* genes were downregulated in the SBF material, however, most of the *SAUR* genes were downregulated in the MBF material, it indicated that the *SAUR* genes expression differences might cause morphological differences, and it could positively regulate the regenerated seedling from SBF material to grow along the longitudinal axis.

Considering the research on phytohormone cytokinin, in *Arabidopsis thaliana*, the deletion mutation of the cytokinin receptors *AHK2* and *AHK3* genes could significantly reduce the ability of organogenesis from adventitious buds [29]. Our study showed that histidine kinase genes (*HK3* and *HK4*) had higher expression in the two regenerated materials, compared to initial material, suggesting that this class of genes might activate the regenerative capacity of rose somatic embryos. Additionally, overexpression of the transcription regulators type-AARRs gene could reduce the organogenesis of adventitious buds [30]. However, the dual regulation of WUS transcription by type-BARRs was required to maintain plant shoot apical meristematic organization [31]. In this study, expression patterns of these related genes for cytokinin signal transduction were also different, type-AARRs were upregulated in the initial material, and type-BARRs were upregulated in the expression of the two regenerated materials.

The Brassinosteroid (BR) is a naturally occurring plant steroid hormone, which is commonly involved in the regulation of numerous life processes, including cell elongation, cell division and differentiation. BR could promote somatic embryo maturation of cotton [32], and the BR responsive *XTH* genes were involved in internode elongation in adzuki bean [33]. In the previous research, BR could increase the ductility of cell wall by regulating the activity of XTH enzyme. In this study, most of the cell wall modifying enzyme genes, *XTHs* were specifically upregulated in the SBF type, compared with the CK material and MBF material. It indicated that the higher expression of *XTH* genes family members, which might have influence on structural changing in the cell wall, contributing to cell wall expansion and stem internode elongation in the SBF material.

Ethylene is a gaseous planthormone that play a key role in many processes, including seed germination, leaf senescence, fruit ripening, abscission and responses to abiotic and biotic stresses. In the function research on ethylene-related genes, the ethylene-associated mutants exhibited higher bud regeneration efficiency [34]. In the study on regeneration of barley, it had been shown that the use of silver nitrate to block the action of ethylene improved barley callus regeneration [35]. Early studies showed that overexpression of the ethylene transduction genes *(EBF1/2)* could be sufficient to render plants insensitive to ethylene [36]. The ethylene-responsive transcription factor (ERF1/2) could inhibit the expression of the key enzyme genes of ethylene biosynthesis and negatively control ethylene biosynthesis and perception [37–38]. Importantly, the increased induction of somatic embryos from Medicago truncatula embryogenic callus was observed ACC and MGBG treatments. However, in the presence of ethylene inhibitors, including silver nitrate and AVG, these modulators negatively affected somatic embryo formation, further development, and maturation [39]. In this study, the *ERF1/2* genes were substantially downregulated in the initial material, and upregulated in the two regenerated materials, especially in the MBF material of *Rosa hybrida* ‘John F. Kennedy’. Additionally, we found that the expression levels of ethylene transduction genes *(EBF1/2)* were substantially upregulated in the MBF material. On the contrary, the highly expression of *EBF1/2* could weaken the sensitivity of the MBF material to ethylene, and made MBF material rarely affected by ethylene, indicating that ethylene might play an inhibitory role in the regeneration process of the MBF material of *Rosa hybrida* ‘John F. Kennedy’.

According to the phytohormone pathways analysis, compared with initial material of somatic embryo, the auxin-responsive factors SAUR and the cell wall modifying enzyme gene *XTH* were upregulated for expression but the protein phosphatase gene *PP2C* was downregulated for expression in SBF type; the higher expression of the ethylene receptor ETR, the ethylene transduction gene *EBF1/2*, the transcription factor EIN3, and the ethylene-responsive transcription factor ERF1/2 were induced by MBF type. It indicated that the significantly differential expression of these phytohormone-related genes might be causing somatic embryo explant to regenerate plantlet under different approaches. In this study, cotyledonary somatic embryos of *Rosa hybrida* ‘John F. Kennedy’ not only could regenerate *via* classic embryogenesis but also organogenesis.

### Function and status of somatic embryo in the process of plantlet regeneration in vitro culture

According to the results of this study, SBF regeneration approach (seed-like germination) and MBF regeneration approach (adventitious buds induction) could be observed on the explants from the same one somatic embryos culture in *Rosa hybrida* ‘John F. Kennedy’, and the ways to regenerate a single bud or multiple buds from SPC explants would be truly different plantlet regeneration patterns from the transcriptome analysis. In our previous study about tissue culture for *Rosa hybrida* ‘John F. Kennedy’ [23], it showed that, based on somatic embryo structure as an intermediate material that could develop in various ways, there might be no significant difference between somatic embryogenesis and organogenesis except whether plantlets were regenerated *via* somatic embryo germination like seeds (classic somatic embryogenesis). Therefore, cotyledonary somatic embryo in *Rosa hybrida* ‘John F. Kennedy’ could be one kind of intermediate material similar to callus in the process of in vitro tissue culture, and then SBF and MBF of plantlet regeneration types could be considered as the simulation of seed propagation and vegetative propagation in the nature.

On the basis of previous studies and our research results, it could be summarized that approaches for plant cell fulfilling totipotency were as follows: plantlet regeneration was developed mainly through organogenesis (the simulation of vegetative propagation in the nature); in the process of plant tissue culture, the different organs (adventitious bud or adventitious root) or various intermediate materials derived from the explants often occurred; and there were three main types about intermediate materials, including callus [9], protocorm-like bodies (PLBs) [40] and embryoid bodies (somatic embryos) [41]. Additionally, adventitious buds or roots [42], even somatic embryos [43] could be induced from callus, and the protocorm-like bodies were metamorphosis stems actually. Only the regeneration approach *via* somatic embryo germination like seeds could be recognized as the classic somatic embryogenesis.

**Fig. 8:**
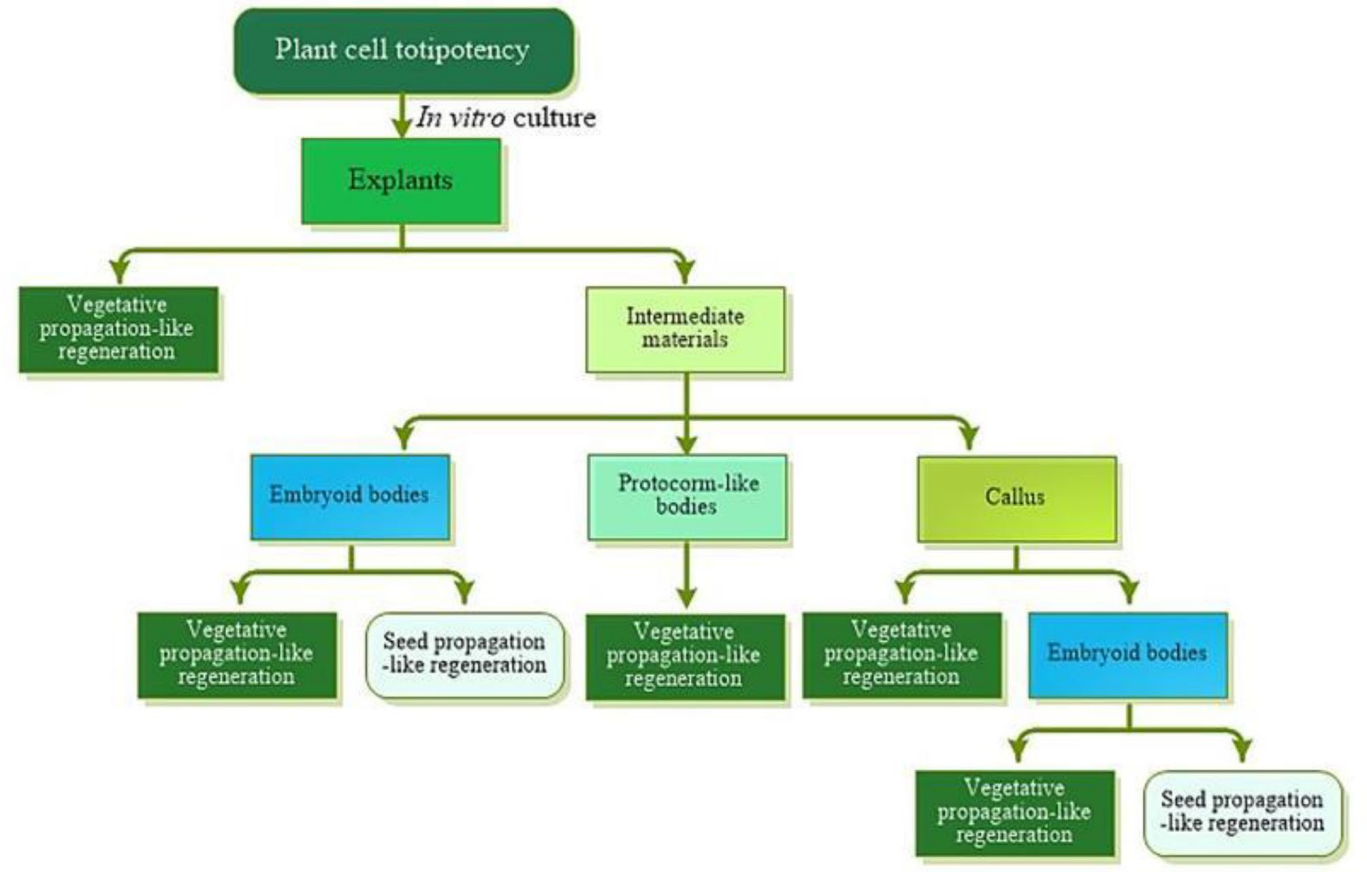
Schematic representation of the procedure of plant cell fulfilling totipotency

Seeing that somatic embryo could be an intermediate material like callus of in vitro culture, not an indicator material for somatic embryogenesis, except that somatic embryo explant germinated like seed in quite low frequency simulating of seed propagation in the nature, the explant could regenerate plantlet mainly through organogenesis approach simulating of vegetative propagation in the nature; the other regeneration approaches might be mostly the metamorphosis of organogenesis actually. The procedure of plant cell fulfilling totipotency represented in Fig. 8.

## Conclusions

According to the GO and KEGG analysis, it indicated the DEGs between two different regenerated materials from somatic embryos were significantly different which might be causing morphological differences. That was somatic embryos from *Rosa hybrida* ‘John F. Kennedy’ could regenerate plantlet *via* both classic somatic embryogenesis (seed-like germination) and organogenesis, cotyledonary somatic embryos should be considered as one kind of intermediate materials similiar to callus, rather than the indicator materials for somatic embryogenesis.

## Materials and methods

### Plant materials and culture conditions

The *Rosa hybrida* ‘John F. Kennedy’ in this study was taken from the rose germplasm resources nursery of the Nanyang Academy of Agricultural Sciences in Henan Province. The annual branches from *Rosa hybrida* ‘John F. Kennedy’ were cut into 1.0 to 1.5 cm stem segments with an axillary bud and sterilized with disinfectant mercuric chloride (0.1% (v/v) HgCl_2_) (HgCl_2_; Tongxin; Guizhou; China). Subsequently, the sterilized materials were placed on MS medium (Solarbio; Beijing; China) supplemented with 87.64 mM sucrose (Kemiou; Tianjin; China), 23.79 mM agar (Biosharp; Hefei; China), 4.44 × 10^^^-3 mM 6-BA (Solarbio; Beijing; China), and 4.92 × 10^^^-4 mM IBA (IBA; Solarbio; Beijing; China) to obtain sterile seedlings. Cotyledonary somatic embryos (CSE) induction and propagation of *Rosa hybrida* ‘John F. Kennedy’ were carried out by following the procedure described previously by Zhu. (2022) [23], which these initial materials were at the same genetic background from the clonal line of Rose ‘KND-3’. Briefly, on MS medium containing a high concentration of 2,4-D (3.17 × 10^-2 mM) (Solarbio; Beijing; China) in the dark, the leaves of sterile seedlings formed embryogenic callus (EC). The EC were put on MS medium with 4.44 × 10^^^-3 mM 6-BA, 5.37 × 10^^^-4 mM NAA (Solarbio; Beijing; China), and 2.89 × 10^^^-4 mM GA_3_ (Solarbio; Beijing; China), and, after a period of time, cotyledonary somatic embryos (CSE) were induced. CSE explants were subcultured to P medium (MS + 2.22 × 10^^^-3 mM 6-BA + 4.92 × 10^^^-4 mM IBA + 87.64 mM sucrose + 23.79 mM agar) for propagation every 30 days. Complete single-piece cotyledonary somatic embryo explants (SPC explants) were selected from proliferated CSE and cultured on P medium to preparation for the samples of transcriptome sequencing. The cultures were incubated at 24 ± 2 °C with a daily 16-h illumination regimen of 2000 Lx light intensity provided by white fluorescent lights unless otherwise specified.

### Samples and RNA isolation

After the 35 days culture of SPC explants (SPC culture), the representative samples were separated and obtained from different embryonic cultures, followed by use of 1×PBS sterile buffer cleaning the sample surface, then quickly frozen in liquid nitrogen for 1 h and stored at -80 °C for RNA isolation. RNA from two types of regenerated samples which were removed non-study tissue sites, were respectively, SBF (single bud formed from SPC explants, Fig. 1c) type and MBF (multiple buds formed from SPC explants, Fig. 1e) type with the 0 d SPC explants (sample of initial material, Fig. 1b) being used as the control (CK), and then separately submitted for transcriptome sequencing with three biological replicates each type. At last, we performed the 2 × 150 bp paired-end sequencing (PE150) on an illumina Novaseq™ 6000 (LC-Bio Technology CO., Ltd., Hangzhou, China) for RNA libraries.

### Transcriptome sequencing and data analysis

To obtain high-quality clean reads, low-quality reads, reads with *N* > 5%, adapter-polluted reads, and reads containing polyA and polyG were removed by filtering with Cutadapt [44]. We aligned reads of all samples to the reference genome using Hisat2 (https://daehwankimlab.github.io/hisat2/, version: hisat-2-2.2.1) [45], and then maps the reads to the reference genome. The mapped reads of each sample were assembled using StringTie (http://ccb.jhu.edu/software/stringtie/, version: Stringtie-2.1.6) [46] with default parameters. After the final transcriptome was generated, gene expression levels of all transcripts were calculated with StringTie and ballgown (http://www.bioconductor.org/packages/release/bioc/html/ballgown.html). The expression abundance of mRNAs was performed by calculating the FPKM (fragments per kilobase per transcript per million mapped reads) value. Genes differential expression analysis was performed by DESeq2 (http://www.bioconductor.org/packages/release/bioc/html/DESeq2.html) [47] software between two different groups (and by edger [https://bioconductor.org/packages/release/bioc/html/edgeR.html] [48] between two samples). The genes with the parameter of false discovery rate (FDR) below 0.05 and absolute fold change (FC) ≥ 2 were considered differentially expressed genes [49]. Annotation of gene ontologies and enrichment analysis of metabolic pathways were based on the Gene Ontology Database (http://www.geneontology.org/) [50] and the Kyoto Encyclopedia of Genes and Genomes (KEGG) pathway (https://www.kegg.jp/kegg/) [51], respectively.

### Differential gene expression validation by RT-qPCR

To verify the reliability of the RNA-Seq data, five differentially expressed genes (LOC112175470, LOC112164757, LOC112189201, LOC112182880, and LOC112182926) were randomly selected for quantitative real-time PCR analysis. The corresponding primer synthesis information is listed in Table 3. The amplification program was performed according to the following scheme: 39 cycles of 95 °C for 3 min, 95 °C for 10 s, and 60 °C for 30 s, followed by the generation of melting curves for verification of amplification specificity, with three replicates of all reactions. Statistical analysis was performed using the 2^−ΔΔCT^ method.

**Table 3:** Primer information

Gene Number	Primer Name	Sequences(5’to 3’)	Annealing Temperature TM
1	18 S -F	ATTCTATGGGTGGTGGTGCAT	60
	18 S -R	TCAAACTTCGCGGCCTAAA	
2	> LOC112175470-F	CCAGTCTTGTTCTTCTCTATGTC	60
	> LOC112175470-R	CATGTCATTCCCGTACTTCTC	
3	> LOC112164757-F	AGACGAGGAGCAAGCATC	60
	> LOC112164757-R	CGAAACCAACACAACGAGAA	
4	> LOC112182880-F	CTGGAGCCTAAACTTTCAAGATC	60
	> LOC112182880-R	CCCATCACTCATCAAACCTTAC	
5	> LOC112189201-F	TGAACCACATGATCTACGA	60
	> LOC112189201-R	CTTCACCCAGAATCAGTATG	
6	> LOC112182926-F	CCATCATCTTCAACAAGGACAG	60
	> LOC112182926-R	GATTTGAAAGGAATGGCTCTGA	

## Data Availability

The reference genome information of Rose chinensis RchiOBHm-V2 was obtained from NCBI (https://ftp.ncbi.nlm.nih.gov/genomes/all/GCA/002/994/745/GCA_002994745.2_RchiOBHm-V2/). All data generated or analysed during this study are included in this published article. The datasets used and/or analysed during the current study are available from the corresponding author on reasonable request.

## References

[1] Hibrand Saint-Oyant L, Ruttink T, Hamama L (2018). A high-quality genome sequence of *Rosa chinensis* to elucidate ornamental traits. Nat Plants.

[2] Gao L, Bao M (2005). Optimization of Agrobacterium-mediated transformation of *Rosa Hybrida*. J Beijing Forestry Univ.

[3] Parth D, Shikha D, Rutul R, Ghanshyam P. Chapter 5-Plant tissue culture: somatic embryogenesis and organogenesis. Adv Plant Tissue Cult. 2022;109–30. 10.1016/B978-0-323-90795-8.00006-0

[4] Bao Y, Liu G, Shi X (2012). Primary and repetitive secondary somatic embryogenesis in *Rosa hybrida*‘Samantha’. Plant Cell Tiss Organ Cult.

[5] Zhang M, Wang A, Qin M, Qin X, Yang S, Su S, Sun Y, Zhang L (2021). Direct and indirect somatic embryogenesis induction in *Camellia Oleifera* Abel. Front Plant Sci.

[6] Hsieh YF, Jain M, Wang J (2017). Direct organogenesis from cotyledonary node explants suitable for Agrobacterium-mediated transformation in peanut (*Arachis hypogaea* L). Plant Cell Tiss Organ Cult.

[7] Ishizaki T (2021). A tissue culture system for callus formation and plant regeneration using tuber discs of *Dioscorea Tokoro* Makino, a pharmaceutical yam species. Biologia.

[8] Ramírez-Mosqueda MA, Iglesias-Andreu LG (2015). Indirect organogenesis and assessment of somaclonal variation in plantlets of *Vanilla planifolia*Jacks. Plant Cell Tiss Organ Cult.

[9] Kshirsagar PR, Mohite A, Suryawanshi S (2021). Plant regeneration through direct and indirect organogenesis, phyto-molecular profiles, antioxidant properties and swertiamarin production in elicitated cell suspension cultures of *Swertia minor* (Griseb.) Knobl. Plant Cell Tiss Organ Cult.

[10] Shen H, Chen J, Chung H (2018). Plant regeneration via direct somatic embryogenesis from leaf explants of *Tolumnia* Louise Elmore ‘Elsa’. Bot Stud.

[11] Kessel-Domini A, Pérez-Brito D, Guzmán-Antonio A, Barredo-Pool FA, Mijangos-Cortés JO, Iglesias-Andreu LG, Cortés-Velázquez A, Canto-Flick A, Avilés-Viñas SA, Rodríguez-Llanes Y (2022). Indirect somatic embryogenesis: an efficient and genetically Reliable Clonal Propagation System for *Ananas comosus* L. Merr. Hybrid MD2. Agriculture.

[12] Zobrist JD, Martin-Ortigosa S, Lee K, Azanu MK, Ji Q, Wang K. Transformation of Teosinte (Zea mays ssp. parviglumis) *via* Biolistic bombardment of seedling-derived callus tissues. Front Plant Sci. 2021;12. 10.3389/fpls.2021.773419 10.3389/fpls.2021.773419 PMC8696365 34956270

[13] Du D, Jin R, Guo J, Zhang F (2019). Infection of embryonic callus with *Agrobacterium* enables High-Speed Transformation of Maize. Int J Mol Sci.

[14] Capote T, Usié A, Barbosa P (2019). Transcriptome dynamics of cork oak (*Quercus suber*) somatic embryogenesis reveals active gene players in transcription regulation and phytohormone homeostasis of embryo development. Tree Genet Genomes.

[15] Guo H, Guo H, Zhang L, Tang Z, Yu X, Wu J, Zeng F (2019). Metabolome and transcriptome association analysis reveals dynamic regulation of purine metabolism and flavonoid synthesis in transdifferentiation during somatic embryogenesis in cotton. Int J Mol Sci.

[16] Reuther J, Roy A, Monzon FA. Transcriptome Sequencing (RNA-Seq). In: Netto, G., Kaul, K, editors Genomic Applications in Pathology. 2019;33–49. 10.1007/978-3-319-96830-8_4

[17] Tang J, Wang FY, Zhang L (2023). Transcriptome analysis based on character difference between Shatang tangerine and Lantern Shatang tangerine. J Fruit Sci.

[18] Ge H, Song W, Liu X (2022). Study on color and transcriptome of backcross branches of *Salix Aureus*. Forestry Ecol Sci.

[19] Li Q, Gao C, Xu K (2021). Transcriptome-based analysis of resistance mechanism to black point caused by *Bipolaris sorokiniana* in wheat. Sci Rep.

[20] Lu J, Zhang Q, Lang L (2021). Integrated metabolome and transcriptome analysis of the anthocyanin biosynthetic pathway in relation to color mutation in miniature roses. BMC Plant Biol.

[21] Yu CX. Somatic embryo induction and its transcriptome analysis of rose. 2021. M. D. Dissertation. Yunnan University, Kunming, Yunnan, China. (in Chinese).

[22] Zhang X, Wang Y, Yan Y (2019). Transcriptome sequencing analysis of maize embryonic callus during early redifferentiation. BMC Genomics.

[23] Zhu ZF. Studies on plant regeneration *via* somatic embryogenesis and its characters in physiology and biochemistry of *Rosa hybrida* L. 2022. M. D. Dissertation. Nanyang Normal University, Nanyang, Henan, China. (in Chinese).

[24] Du L, Kang XL, Zhu ZF et al. Plantlet regeneration *via* somatic embryogenesis and changes in endogenous hormone content of *Rosa hybrida* ‘J. F. Kennedy’. 2023. (In press).

[25] Su Y, Zhao X, Liu Y, Zhang C, O’Neill SD, Zhang X (2009). Auxin-induced WUS expression is essential for embryonic stem cell renewal during somatic embryogenesis in Arabidopsis. Plant J.

[26] Cheng Y, Dai X, Zhao Y (2007). Auxin synthesized by the YUCCA flavin monooxygenases is essential for embryogenesis and leaf formation in *Arabidopsis*. Plant Cell.

[27] Zhao Y (2012). Auxin biosynthesis: a simple two-step pathway converts tryptophan to indole-3-acetic acid in plants. Mol Plant.

[28] Niek S, Marian B (2019). The *SAUR* gene family: the plant’s toolbox for adaptation of growth and development. J Exp Bot.

[29] Hguchi M, Pischke MS, Mahonen AP, Miyawaki K, Hashimoto Y, Seki M, Kobayashi M, Shinozaki K, Kato T, Tabata S, Helariutta Y, Sussman MR, Kakimoto T (2004). In planta functions of the Arabidopsis cytokinin receptor family. P Natl Acad Sci USA.

[30] Buechel S, Leibfried A, To JEC, Zhao Z, Andersen SU, Kieber JJ, Lohmann JU (2010). Role of A-type ARABIDOPSIS RESPONSE REGULATORS in meristem maintenance and regeneration. Eur J Cell Biol.

[31] Meng W, Cheng Z, Sang Y, Zhang M, Rong X, Wang Z, Tang Y, Zhang X (2017). Type-B ARABIDOPSIS RESPONSE REGULATORs specify the shoot stem cell niche by dual regulation of WUSCHEL. Plant Cell.

[32] Aydin Y, Ogras T, Altınkut A, İsmailoğlu I, Arican E, Gozukirmizi N (2010). Cytohistological studies during cotton somatic embryogenesis with Brassinosteroid Application. IUFS J Biology.

[33] Nakamura T, Yokoyama R, Tomita E, Nishitani K (2003). Two azuki bean XTH genes, *VaXTH1* and *VaXTH2*, with similar tissue-specific expression profiles, are differently regulated by auxin. Plant Cell Physiol.

[34] Chatfield SP, Raizada MN (2008). Ethylene and shoot regeneration: hookless1 modulates de novo shoot organogenesis in *Arabidopsis thaliana*. Plant Cell Rep.

[35] Jha AK, Dahleen LS, Suttle JC (2007). Ethylene influences green plant regeneration from barley callus. Plant Cell Rep.

[36] Guo H, Ecker JR (2003). Plant responses to ethylene gas are mediated by SCFEBF1/EBF2-dependent proteolysis of EIN3 transcription factor. Cell.

[37] Xu S, Geng X, Wang L (2021). A review of the structure, function and expression regulation of ethylene response factors (ERF) in plant. J Zhejiang A&F Univ.

[38] Nowak K, Wojcikowska B, Gaj MD (2015). *ERF022* impacts the induction of somatic embryogenesisin Arabidopsis through the ethylene-related pathway. Planta.

[39] Mantiri FR, Kurdyukov S, Lohar DP, Sharopova N, Saeed NA, Wang X, Vandenbosch KA, Rose RJ (2008). The transcription factor MtSERF1 of the ERF subfamily identified by transcriptional profiling is required for somatic embryogenesis induced by auxin plus cytokinin in *Medicago truncatula*. Plant Physiol.

[40] Tian C, Chen Y, Zhao X (2008). Plant regeneration through protocorm-like bodies induced from rhizoids using leaf explants of Rosa spp. Plant Cell Rep.

[41] Xu Kd, Wang W, Yu D (2019). NAA at a high concentration promotes efficient plant regeneration via direct somatic embryogenesis and SE-mediated transformation system in *Ranunculus sceleratus*. Sci Rep.

[42] Hu G, Chao M, Fu Y (2022). In vitro protocol for bud induction from adventitious roots and hydroponic acclimatization of purple sweet potato (*Ipomoea batatas* (L.) Lam). Plant Cell Tiss Organ Cult.

[43] Liu Y, Wei C, Wang H (2021). Indirect somatic embryogenesis and regeneration of *Fraxinus mandshurica* plants via callus tissue. J Res.

[44] Martin M (2011). Cutadapt removes adapter sequences from high-throughput sequencing reads. EMBnet J.

[45] Kim D, Paggi JM, Park C (2019). Graph-based genome alignment and genotyping with HISAT2 and HISAT-genotype. Nat Biotechnol.

[46] Kovaka S, Zimin AV, Pertea GM, Razaghi R, Salzberg SL, Pertea M (2019). Transcriptome assembly from long-read RNA-seq alignments with StringTie2. Genome Biol.

[47] Love MI, Huber W, Anders S (2014). Moderated estimation of Fold change and dispersion for RNA-seq data with DESeq2. Genome Biol.

[48] Robinson MD, McCarthy DJ, Smyth GK (2010). edgeR: a Bioconductor package for differential expression analysis of digital gene expression data. Bioinformatics.

[49] Benjamini Y, Hochberg Y (1995). Controlling the false Discovery rate: a practical and powerful Approach to multiple testing. J Royal Stat Soc Ser b-Methodological.

[50] Carbon S, Douglass E, Good BM (2021). The Gene Ontology resource: enriching a GOld mine. Nucleic Acids Res.

[51] Kanehisa M, Furumichi M, Sato Y, Ishiguro-Watanabe M, Tanabe M (2021). KEGG: integrating viruses and cellular organisms. Nucleic Acids Res.

